# PET/MRI: a frontier in era of complementary hybrid imaging

**DOI:** 10.1186/s41824-018-0030-6

**Published:** 2018-06-25

**Authors:** Sikkandhar Musafargani, Krishna Kanta Ghosh, Sachin Mishra, Pachaiyappan Mahalakshmi, Parasuraman Padmanabhan, Balázs Gulyás

**Affiliations:** 1 Lee Kong Chian School of Medicine, Nanyang Technological University, 59 Nanyang Drive, Singapore, 636921 Singapore; 2 School of Electrical Engineering, Vellore Institute of Technology, Vellore, 632014 India

**Keywords:** Multi-modal imaging, Hybrid modality, PET/MRI, Attenuation correction, Image reconstruction

## Abstract

With primitive approaches, the diagnosis and therapy were operated at the cellular, molecular, or even at the genetic level. As the diagnostic techniques are more concentrated towards molecular level, multi modal imaging becomes specifically essential. Multi-modal imaging has extensive applications in clinical as well as in pre-clinical studies. Positron Emission Tomography (PET) has flourished in the field of nuclear medicine, which has motivated it to fuse with Computed Tomography (CT) and Magnetic Resonance Imaging (MRI) for PET/CT and PET/MRI respectively. However, the challenges in PET/CT are due to the inability of simultaneous acquisition and reduced soft tissue contrast, which has led to the development of PET/MRI. Also, MRI offers the better soft tissue contrast over CT. Hence, fusion of PET and MRI results in combining structural information with functional image from PET. Yet, it has many technical challenges due to the interference between the modalities. Also, it must be resolved with various approaches for addressing the shortcomings of each system and improvise on the image quantification system. This review elaborates on the various challenges in the present PET/MRI system and the future directions of the hybrid modality. Also, the different data acquisition and analysis techniques of PET/MRI system are discussed with enhanced details on the software tools.

## Background

Molecular imaging is the visualisation, characterisation and measurement of biochemical process at the molecular and cellular level in living organisms. It could be either functional such as PET, functional MRI (fMRI), Magnetic Resonance Spectroscopy (MRS) or anatomical such as CT or MRI. This is used for early assessment, risk stratification, evaluation and follow-up of patients with cardiovascular, oncological and neurological diseases. The available technologies may vary in spatial resolution, depth penetration, expenditure of energy over image formation, biocompatibility of the injectable molecular probes and detecting limitation factors of the molecular probe. Each of these techniques has its own merits and demerits over the other. Hence, there is a need for the fusion of two or more techniques to yield the complementary information which the other could not afford to provide. It is known as Multi-Modality Imaging (MMI) technique (Martí-Bonmatí et al. 2010).

PET is a non-invasive metabolic imaging technique which traces the physiological and pathophysiological process at molecular level (Boss et al. 2010). This technique involves the principle of nuclear imaging as it employs radiotracers. It is based on the positron decay and annihilation, which yields a pair of gamma rays being separated at an angle of 180 degree, that could be captured on collinearly aligned detectors such as scintillators (Townsend 2004). The pattern of electronic collimation makes it more sensitive among all the nuclear imaging technique. However, PET does not offer any anatomical information, which turns out to be the major drawback of it.

On the other hand, MRI offers an immense 3-D soft tissue contrast with the specified levels of T1 and T2 relaxation time constants. This enables it to be a privileged technique as a first-line method of choice for the assessment of tumour. The magnetic field strength of scanners for human is in the range of Ultra High Frequency (UHF). It provides the structural imaging with higher spatial resolution and functional (Blood Oxygen Level Dependent) contrast (Olman et al. 2012). Also, the columnar level of resolution increases the contrast of the image. The functional imaging by MRI includes Diffusion Weighted Imaging (DWI), MRSand Perfusion Weighted Imaging (PWI). Thus, providing high end accuracy of estimation in tumour grading (Fink et al. 2015; Holdsworth and Bammer 2008). However, the sensitivity and specificity of the functional information provided by MRI is minimal compared to that of PET. In other way around, MRI technique sounds meritorious over CT as it is devoid of harmful radiations. The additional advantage of MRI is that it offers motion correction in reconstruction of the anatomical information (Atkinson 2011).

The recent researches in clinical and preclinical imaging trials employ PET and MRI extensively, irrespective of the shortcomings of either of them. Hence, the combination of these two modalities could produce great soft tissue contrast, unique flexibility in acquisition parameters for characterising the tissue, minimised exposure to radiation with enhanced sensitivity for desired clinical and research applications (Boss et al. 2010). PET/MRI was introduced in the year 1990, along with the proposal of PET/CT. But, PET/CT flourished sooner than PET/MRI due to the various technical challenges involved in combining PET and MRI as a hybrid modality. Beyond the magnificent applications of PET/CT, it has acquired the shortcomings that overshadow the imaging modality with PET/MRI (Paspulati et al. 2015). This leads to the opening up of an era with multifunctional and multi-parametric imaging. In contrast to PET/CT, PET/MRI has superior soft tissue contrast, low dosage of radiations and could be performed on any part of the body. The low dosage seems to be advantageous in small animal imaging for serial and longitudinal studies (Xu 2014). The major suppressing fact of PET/CT is the sequential imaging design. It involves acquisition from the two systems followed by attenuation correction in the CT information through software modules and causes errors due to patient motion between the two acquisitions. It is very hectic due to lengthy scanning time. On the other hand, the later has motion correction through MRI information. Thus, the PET/MRI turns out to be a potent in many clinical and pre-clinical applications with substantiating merits. The clinical applications have gone through wide edges in the field of oncology, cardiology, neurology and musculoskeletal.

This review work discusses upon the design of PET/MRI and revolves around the challenges in meeting the fusion of PET with MRI and the evolution of the system over years. Further, it discusses about the image acquisition with attenuation correction and reconstruction methods to produce a quantitative image. It also magnifies and contributes to the current challenges and future directions of PET/MRI.

### Physical challenges in integrating PET and MRI

**Fig. 1:**
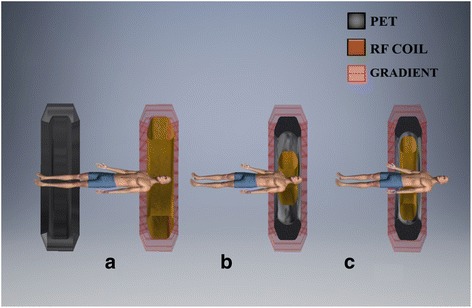
Shows the different configurations of PET/MRI (**a**) Sequential design, (**b**) Simultaneous PET insert MRI scanner and (**c**) Simultaneous fully integrated system

**Fig. 2:**
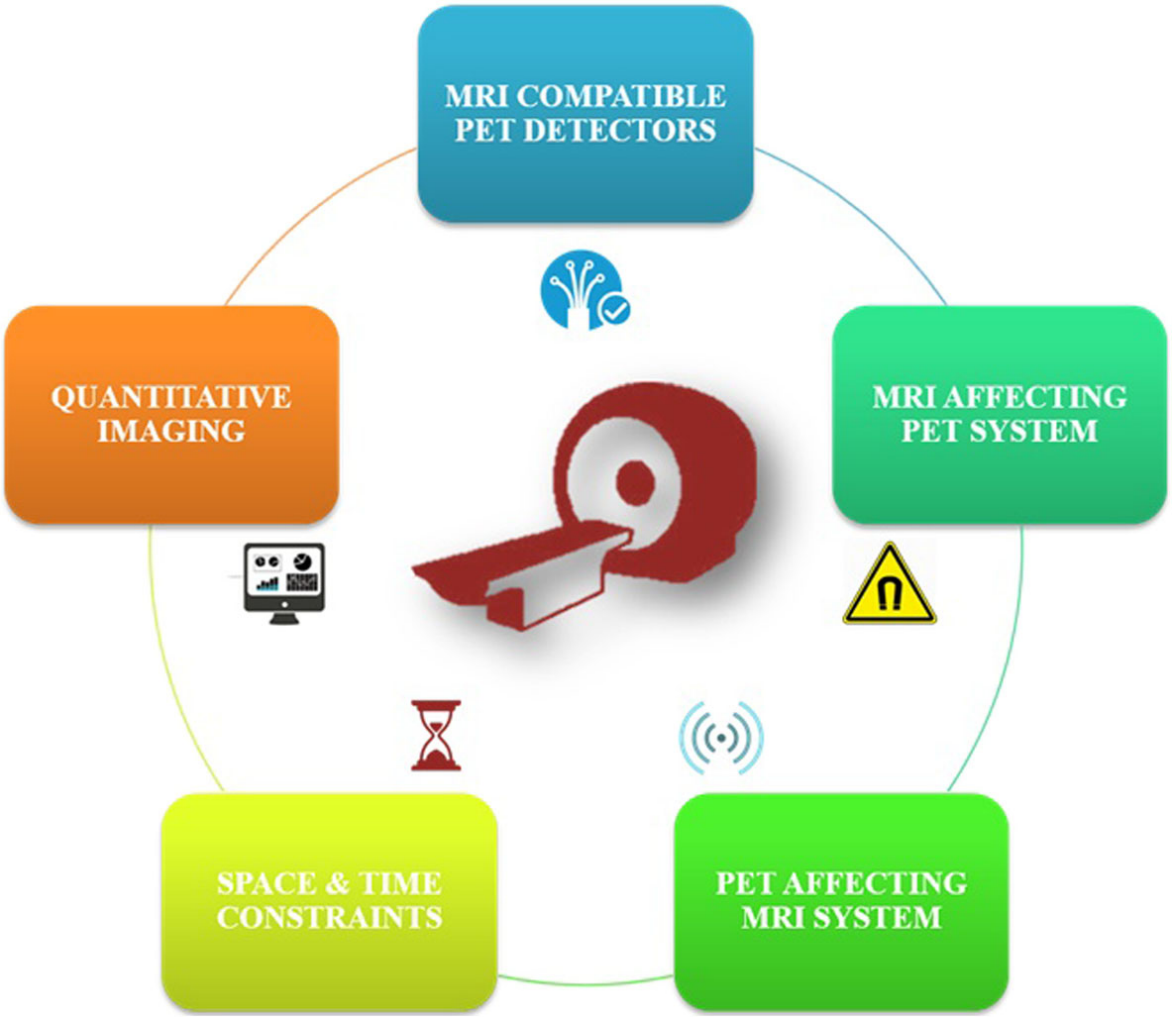
Challenges in fusing PET and MRI

The design of PET-MRI is complex due to the technical challenges caused by the presence of magnetic field. A fully integrated system must be obtained without compromising on the performance of either of the stand-alone modalities. Besides the software aided post-processing, the primarily used designs are with Sequential and Simultaneous acquisition approach. Figure 1a shows the sequential (tandem) configuration in which the PET and MRI are taken one after the other in a sequence followed by software deployed co-registration**.** The dual approach for this design is same patient transfer table top and same bed for the patients. The former approach is employed when the modalities are housed in the same room, while the latter is for different compartments. The sequential design technique is assumed to be economical, simplex design, minimised claustrophobia due to separated modalities, minimal influence of magnetic field with additional shielding, etc. (Cho et al. 2007).It is adopted by Philips Healthcare, which is known to be TF-PET/MRI (Zaidi et al. 2011). However, it introduces motion artefacts of the organs and occupies large space to accommodate the huge instrumentation of PET/MRI, which typically requires a room space of 4.3 × 13 m (Zaidi and Del 2011). Hence, it justifies on the need for concurrent imaging modality. The simultaneous acquisition design has an objective to build both the modalities within each other, i.e., to share a common gantry for both the imaging modalities. It exists for usage since 2006. Thus, it could be obtained in two modes: PET insert MRI scanner (Fig. 1b) and fully integrated system (Fig. 1c). This approach is quite prevailing as it aims to minimise the occupancy of the system. On the other hand, the design should resolve numerous technical obstacles related to the compatibility of the system. Let us discuss the physical challenges in integrating MRI and PET (Fig. 2).

### MRI system affecting PET

Despite the challenges in physical interaction between the two modalities due to the bore size and thickness of block detectors in PET, the main objective is to achieve the high performance of both without compromising on each of them. The functionality of PET has been affected due to very high static magnetic field, gradient magnetic field and Radiofrequency signals (Cumalat et al. 1990) (Refer Table 1).

**Table 1:** MRI system affecting PET

Components of MRI	Effect on PET	Consequences	Resolution
Static magnetic field (B_0_)	Influences the ray of electron flow in read out.	Lower gain factor.	Substitute the Photomultiplier with Solid State Photo Detector (SSPD) such as SiPM or APD.
Gradient magnetic field (B_1_)	Heats up the equipment leading to vibrations.	Induction of eddy currents and mechanical vibrations.	Restructuring of electronic components. Fixing temperature control device.
RF Interference	Introduces noise signal.	Due to magnetic induction, introduces eddy current. Produces heating effect.	Introducing RF shield around the coil.

#### Static magnetic field

The block detectors present in the scintillator of PET system consists of electron flow in Photo Multiplier Tubes (PMT), which has been perturbed by the very high static magnetic field (Hoult and Phil 2000).The trajectory of electrons between the dynodes get deflected due to Lorentz force and cause the loss of information in a particular region (Yamamoto et al, 2004). Thereby, the PMT tubes have to be shielded by using steel or mu-metals (Junnarkar et al. 2006). Also, the PMT could be replaced with light detectors such as optical fibres that have better performance in the vicinity of magnetic field as it contains only x-rays and gamma rays for detection. However, the length of the optical fibres causes the attenuation of the signal (Slates et al. 2015). Rather, The PMT could be replaced with field insensitive PMTs such as Avalanche Photodiode (APD) and position sensitive PMTs (PSPMT) to make it less sensitive to the extensive magnetic field (Grazioso et al. 2006; Watanabe et al. 1997). APD can be directly connected to the scintillator crystal of block detector with a short light guide of 1–2 mm and it is also insensitive to magnetic field. Although, it is very sensitive to temperature variations and has lower gain factor (Pichler et al. 2004).

#### Gradient magnetic field

The gradient magnetic fields get switched rapidly at a rate of 1 kHz for achieving the greater skin depth at lower frequencies which induces eddy currents within the PET circuitry (Klose 1990). The introduction of eddy currents causes heating up and mechanical vibrations of the electronics present in PET system. Thus, the high frequency electronics is supposed to be shielded with aluminium or copper with connection to the ground. The non-magnetic and non-ferromagnetic nature of copper enables it to provide about 99% of electrical isolation around the electronics of PET detectors (Pichler et al. 2006). Due to the gradient sequence, the sensitivity of the PET/MR system diminishes by 5–20% (Grazioso et al. 2006). The Solid State Photo Detectors and the other electronics present closer to the gradient coil has to be highly robust in nature (Woody et al. 2007). If else, the PET readout system is supposed to be redesigned to damp the oscillations.

#### RF signal

The RF signals generated by transmission coils of MRI cause the susceptibility of the electronics in the PET system during MRI acquisition in simultaneous acquisition modality (Woody et al. 2007). This effect is observed adversely in the high frequency range. The RF interference causes the reduction of SNR. This could be overcome by shielding of PET detectors with conductive materials such as copper. However, it introduces the flow of eddy currents around the conductive shielding. Hence, the RF interference can be suppressed by ‘coupling-decoupling’ of RF receiver coil by varying the clock frequencies and clock phase relations of the digital circuits. Thereby, the emitted RF field can be optimised to work with the lower field coupling into the MRI-RF coil. It was performed using FPGA based frequency and phase switching of diSPM used in the PET modules of Hyperion II^D^ PET ins (Gebhardt et al. 2016).

### PET system affecting MRI

Conversely, the presence of PET detectors within the magnetic bore also affects the magnetic properties of the MRI system due to the following factors (Refer Table 2).

**Table 2:** PET system affecting MRI

Components of PET	Effect on MRI	Consequences	Resolution
Scintillator detector	Non-uniformity of main magnetic field (B_0_)	Introduces magnetic induction	Substituting with MRI compatible scintillation detectors
Gamma Shielding	Introduction of distortion and non-linearity due to eddy currents	Higher cost	Alternative shielding materials are chosen
Electronics and cables of PET	Causes to RF interference	Heating effect	Introducing RF shield around PET detectors

#### Susceptibility artefacts

The introduction of components of PET system within the bore of the magnet affects the uniformity of the magnetic field due to slight variations in the magnetic susceptibility. Thus, it affects the linearity of the gradient field, which could be resolved accurately by shimming (Pichler et al. 1997). *RB Stales* et al. performed an experiment to determine the reason and degree of susceptibility artefacts by T2 weighted imaging of the orange in a small prototype of MR compatible PET scanner (McPET). However, he concluded that the placement of moderate amount of LSO or optical fibres does not produce much changes in the image (Berker et al. 2014). Also, there could be an arise of susceptibility artefacts due to metal implants present in the adjacent lesion in simultaneous TOF-PET/MRI (Zaidi et al. 2011). Besides this, the eddy currents tend to cause distortion of static magnetic field and affects linearity of the gradient field (España et al. 2010). This effect could be set back by using non-magnetic materials in the PET detectors to preserve the homogeneity of the magnetic field.

#### RF interference

MRI scanning intends to obtain the NMR signal as a response to the excitation by gradient field (weak), which requires high sensitive and specific receiver coils. Thereby, Faraday shielding is necessary for MRI scanning room. Also, the clock pulses of modern digital electronics that operates at high frequency are needed to be shielded for preventing interference with magnetic field. Hence, it prevents the non-uniformity of magnetic field and generation of eddy currents around the conductive shield (Yamamoto et al. 2005). In case of asymmetric shielding arrangement with Bruker Birdcage RF coils, which produce the negative impact on the MRI system due to its tight fit (España et al. 2010). Also, the gamma shielding tends to cause the distortion and non-linearity due to the adverse effects of eddy currents (Zaidi et al. 2011). Thus, it makes an essence of RF shielding.

### Space and time constraints

The purpose-designed MRI-compatible PET scanners are housed within the bore of the humungous magnet for acquiring concurrent imaging in many of the human PET-MRI system. The prime factor to be considered is the longer acquisition time of MRI over CT as the former consumes about 20 to 40 min for different imaging sequences, while CT consumes only about 15 s to 1 min for a typical system. The advanced PET technology with 3D scanner, time-of-flight (TOF) and longer axial Field of View (FOV) has higher sensitivity and accuracy. It reduces the acquisition time of brain imaging for 3–15 min and whole body scanning for 10–20 min (Karp et al. 2009). In the sequential acquisition time, the total time for MR and PET imaging is longer, while the simultaneous acquisition time will be dependent on the slowest acquisition modality. On the other hand, the space consideration is necessary for designing a compact system as the sequential acquisition system has to be organised for minimising the occupancy of the standalone systems (Zaidi and Del 2011).

### Various approaches for fusing PET & MRI

**Fig. 3:**
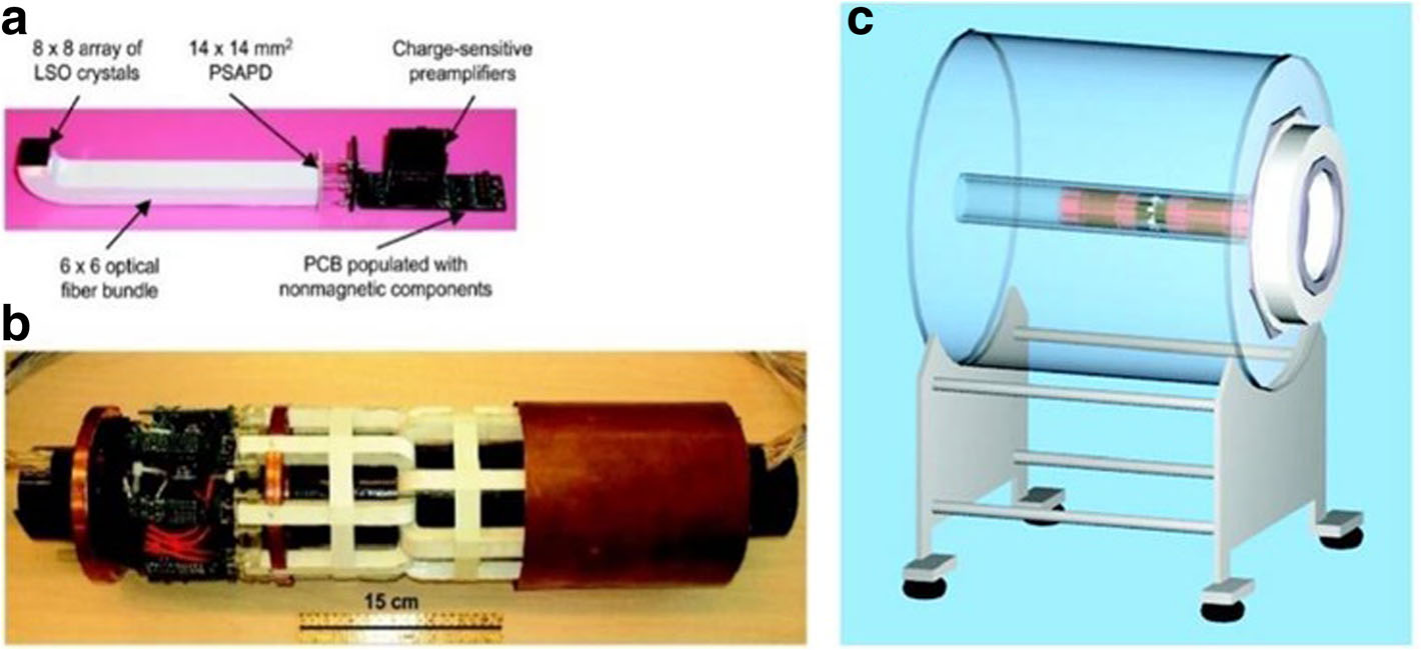
Shows (**a**) MR-compatible PET detector module, (**b**) complete MR-compatible PET scanner with 16 detector modules, shielding material (removed on left side), and carbon fibre tube for support and (**c**) Axial placement of PET insert inside 7 T MRI scanner (This research was originally published in *C. Catana, Y. Wu, M. S. Judenhofer, J. Qi, B. J. Pichler, and S. R. Cherry*, “Simultaneous Acquisition of Multislice PET and MR Images: Initial Results with a MR-Compatible PET Scanner, (Catana et al. 2006)” *J. Nucl. Med.*, vol. 47, no. 12, pp. 1968–1976, 2006.© by the Society of Nuclear Medicine and Molecular Imaging, Inc.)

The best integrated PET-MRI modality looks for MRI compatible PET detectors. The effective detector for gamma rays could be scintillator coupled with light guides and photo detector, which is treated as a standard design (Farahani et al. 1999). Further, the adaptation and enhancement of this standard design could be utilised as an effective design of MRI compatible PET detectors. The usual PET scintillators such as Bismuth Germinate (BGO), Lutetium Oxyorthosilicate (LSO), Lutetium Yttrium Orthosilicate (LYSO), etc. merely affects MRI over Gadolinium based PET scintillators such as Gadolinium Oxyorthosilicate (GSO) and Lutetium Gadolinium Oxyorthosilicate (LGSO), thus, they are seldom used nowadays (Herbert et al. 2006; Kolb et al. 2010). Due to the poor efficiency of PMT in the magnetic field of MRI modality, the preliminary generations of MRI compatible PET detectors aimed to locate the PMTs away from the magnetic field. It was connected through lengthy optical fibres of 1 mm in diameter for directing the optical energy from the scintillator to PMTs and used the light guide for reading out the scintillator (Raylman et al. 2006). The utilisation of large optical fibres leads to the loss in intensity and temporal resolution due to loss of large number of photons (Fig. 3). The attenuation along the length of the fibre introduces transition time and results in slower rise time and diminished temporal resolution (Catana et al. 2006). Hence, it creates a need for substitution of PMTs in block detectors of PET with Solid State Photo Detectors (SSPD), a new era to escalate the performance of PET-MRI.

### Solid state photo detector

A semiconductor device which creates high field region when an external voltage source is connected to it is known as SSPD. It has higher quantum efficiency than PMTs because of the presence of high concentration of electron-hole pairs. They are immune to magnetic field because of the penetration of charge carriers through narrow barrier (Stunkard 2009). The two relevant kinds for PET-MRI system are Avalanche Photodiode (APD) and Silicon Photomultiplier (SiPM). SiPM is also regarded as Geiger mode APD (G-APD) or Multi Pixel Photon Counter (MPPC). The gain of APD saturates at 9.4 T irrespective of orientation (Pichler et al. 1997), whereas this phenomenon could be observed in SiPM at 7 T (España et al. 2010).

#### APD based detectors

**Fig. 4:**
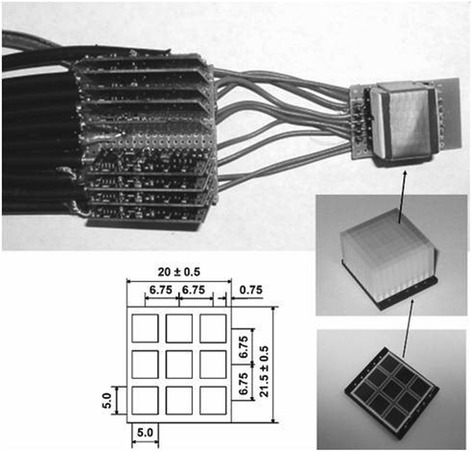
LSO-APD detector assembly: 10 × 10 LSO array (centre right) was coupled by custom-made light guide to 3 × 3 APD array (bottom right) (This research was originally published in *B. J. Pichler* et al.*,* “Performance test of an LSO-APD detector in a 7-T MRI scanner for simultaneous PET/MRI, (Pichler et al. 2006)” J. Nucl. Med., vol. 47, no. 4, pp. 639–647, 2006. © by the Society of Nuclear Medicine and Molecular Imaging, Inc.)

APDs have increased sensitivity towards temperature fluctuations besides its better tolerance against strong magnetic field. Thus, this notion is utilised to attach the detector within the very high magnetic field belonging to MRI set up using a short optical fibre of 1 to 2 mm for preventing the loss of photons. Thereby, it increases the intensity of electrical energy from optical source within the magnetic field. Along these features, APD are smaller size, flexible in design and extends the axial and trans-axial FOV of the PET modality in the hybrid scanner (Fig. 4) (Pichler et al. 2006). The availability of array of detectors provides a large axial FOV for whole body imaging. However, the low gain factor of this detector forces the utilisation of the pre amplifiers and other signal conditioning circuits (Jampel et al. 2009).Also, the interference of electronic noise has to be ceased using copper shielding around it. However, the utilisation of copper shielding involves the effects of eddy currents. Adding up to it, the avalanche multiplication process in APD causes decrease in timing resolution to approximately 1.8 ns, thereby, preventing it to be used with TOF applications (Grazioso et al. 2006). Over these shortcomings of APD, it has been used in many of the modalities in recent times, although SiPM has better performance over it (Nassalski et al. 2008).

#### SiPM based detectors

SiPM works on the principal of Geiger discharge (Popova et al. 2015). It makes a grid of G-APD cells up to 10,000 cells for every mm^2^ in parallel connection. The dynamic range of the detector could be elevated by increasing the number of cells in the specified area. At times, the image gets degraded due to the noise contribution from the dark counts or optical cross over (Otte et al. 2005; Herbert et al. 2006). Apparently, the temperature has an influence over the dark counts of the detector. Precisely, it gets doubled for every rise of 8 degree centigrade (Kolb et al. 2010). The induction of temperature is due to the rapid switching of the gradient magnetic field and RF pulse sequences. Beyond the fall out, the high gain factor and response time with digitized front end electronics in the presence of magnetic field are employed to detect the photons with time resolution of 100 ps full width at half maximum (Llosá et al. 2007). Because of the zero influence of magnetic field over the resolution and intensity, it is more preferred over APD (Herbert et al. 2006). Over the years of advancement in detectors, there have been evolvements in the system design for specific applications in higher order detections.

### Split magnet

An efficacious approach to fit a PET system within a gap on the super conducting magnet of 1 T field strength by Lucas et al. (2006). This is obtained by placing the multi-ring PET detector with 1.2 m long optical fibres between the spaces of 80 mm on the magnet. The PET detector consists of LSO crystals connected with light guides within MRI modalities of PET/MRI system along with the iron shielding around the magnet (Hawkes et al. 2015). The major depleting factor is that optical fibres are left out of the MRI modality, where the magnetic field is diminished to 1 Milli Tesla (mT) at the position PET system. As the probability of light reaching is just 0.4, it degrades the intensity and time resolution. Also, the splitting of magnet diminishes the strength of magnetic field. Rather, the magnetic field strength of greater than 4.7 T is specifically used for small-animal imaging system. Hence, the homogeneity of the magnetic field gets disturbed and retards the gradient performance of the system (Popova et al. 2015).

### Field cycling

The conventional MRI system creates the strong magnetic field which turns out to be the short comings of the system while moving towards to the hybrid scanner system. Hence, the field cycling phenomenon involves the switching between the polarising (strong) and the read out (weak) field. This idea diminishes the need for maintaining the homogeneity of magnetic field evolved. Initially, the polarising field of strength 1 T is set on for a short duration of less than 1 s and turning it off to onset the read out field which is around one-tenth of the polarizing field during which the image is acquired (Handler et al. 2006). Thereby, the contrast of the image could be enhanced by increasing the frequency of switching between the strong and weak field. This phenomenon is employed mainly in small-animal MR scanner. It could also be used in the hybrid system of sequential PET/MRI as a notion to fit in the PET subsystem within the gap of a strong magnet (Gilbert et al. 2006). Also, the concept gets rid of optical fibres with the PMTs in scintillation crystal to reduce the loss of light due to magnetic field (Handler et al. 2006). Due to rapid switching of the magnetic field at high frequency, it tends to deteriorate the functioning of PET sub-modality (Gilbert et al. 2009).

### Brain insert for concurrent imaging

**Fig. 5:**
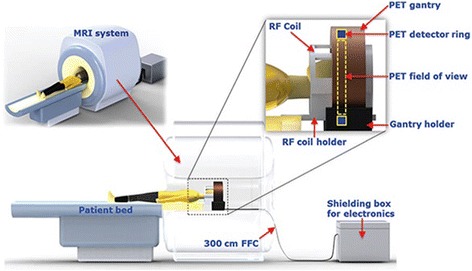
Illustration of PET/MRI integration showing the placement of the PET gantry, RF coil and shielding box (This research was originally published in *K. J. Hong* et al., “A prototype MR insertable brain PET using tileable GAPD arrays, (Hong et al. 2013)” *Med. Phys.*, vol. 40,no. 4, 2013)

The concurrent PET-MR system for imaging the brain was brought up in the year of 2007, where the PET system with RF coil is fitted within the bore of the magnet (Schlemmer et al. 2008). This module of design is adopted by Siemens brain PET insert by using the magnetic field of 3 T (Cherry et al. 2008). The high resolution of the PET detector was acquired by using LSO/APD based detectors which functions as that of the principle in Tubingen small animal system. The detector module is made up of an array of LSO with dimensions of 2.5 × 2.5 × 20 mm^3^ for 12 × 12 array attached with 3 × 3 array light guide of 5x5mm^2^ dimensions (Delso et al. 2011). The utilisation of APDs limits the TOF applications and there is a high demand for faster timing resolution. A quadrature transmitter/receiver circular polarised head coil is used for reducing the gamma photon attenuation connected with the couch of the patient inside the PET. The high sensitivity could be obtained along with the reconstruction techniques of resolution that is less than 3 mm for FWHM (Farahani et al. 1999). The interference of the main magnetic field and gradient field with the functioning of PET is minimum such that it could be used as fMRI and MRS. Although this could provide multidimensional applications, there is an increase in the interference signal due to PET-RF. Thus, it reduces the count rate up to 3% in the standard sequences (Weirich et al. 2012). On the other hand, the sensitivity of the PET detector falls out due to different imaging sequences of MRI, namely, echo planar imaging sequence. The same instrument is applied for the magnetic field strength in the UHF range for examining the human brain at 9.4 T and increases the spatial resolution of the system (Shah et al. 2013). Succeeding it, the *Hong* et al. approached a design which employed the MRI system of 3 T with the detector made up of SiPM with the shielded cables (Fig. 5) (Hong et al. 2013). Thus, the concurrent imaging with this technology led to corruption of image due to RF interference and decreases its SNR due to the eddy currents.

### Sequential & Simultaneous whole-body imaging

The whole-body imaging aims to screen through the entire body for acquiring the various information across the 3-D body. Employment of this hybrid modality could enhance the image features with high spatial and time resolution. This could be either obtained with sequential or simultaneous imaging modality. Although, the sequential system fails to provide high temporal resolution, the designing of the system is quite simpler. Besides, it provides a potential clinical application for multi-purpose imaging such as MRI Attenuation Correction(MRIAC), which is adopted by GE Healthcare (Veit-Haibach et al. 2013). This system employs same couch for both the modality such that MRIAC could be taken with or without MRI coils. If it was taken with MRI, the coil must be taken off prior to the PET imaging. The first commercially available sequential human PET/MRI system is that adopted by Philips Healthcare, which comprises of 3 T whole body MRI and PET satisfying TOF application (Griesmer et al. 2010). The couch could be of sliding type to enable whole body imaging with less scanning time, although most of the electronics of PET is placed external to that of MRI. Thus, it keeps away from RF interference and fringing effect. Also, this could be advantageous to reduce the loss of gain in PMT with the reduction of supply power from 1500 V to DC voltage of 900.Whereas, the simultaneous acquisition modality for whole body imaging consists of the PET detector with annular rings of 56 block detectors and 3 T whole body MRI (Delso et al. 2011). Thereby, it makes the system unfit for TOF application. It has the dimensions of 4x4x20 mm in an array of 3 × 3 providing a temporal resolution of 2.93 ns. The simultaneous imaging puts up an upper limit to fit the ring diameter of trans-axial FOV 59.4 cm within the bore of the MRI subsystem. The MRI subsystem acts like a standalone system for advanced applications like fMRI and MRS. Simultaneous acquisition system is adopted by GE health care for designing TOFPET/MRI system. This model has GE 3 T Discovery 750w MRI system, where the detector is made up of LSO/LYSO with dimensions of 4 × 5.3 × 25 mm (Levin et al. 2013). Another system named as PET NEMA is also introduced with increased sensitivity by recovering the annihilated photons due to Crompton effect (Wagadarikar et al. 2012). The comparable information is acquired for PET NEMA IQ phantom. Thus, attenuation correction and reconstruction of the image must be done.

### Image quantification and its challenges

**Fig. 6:**
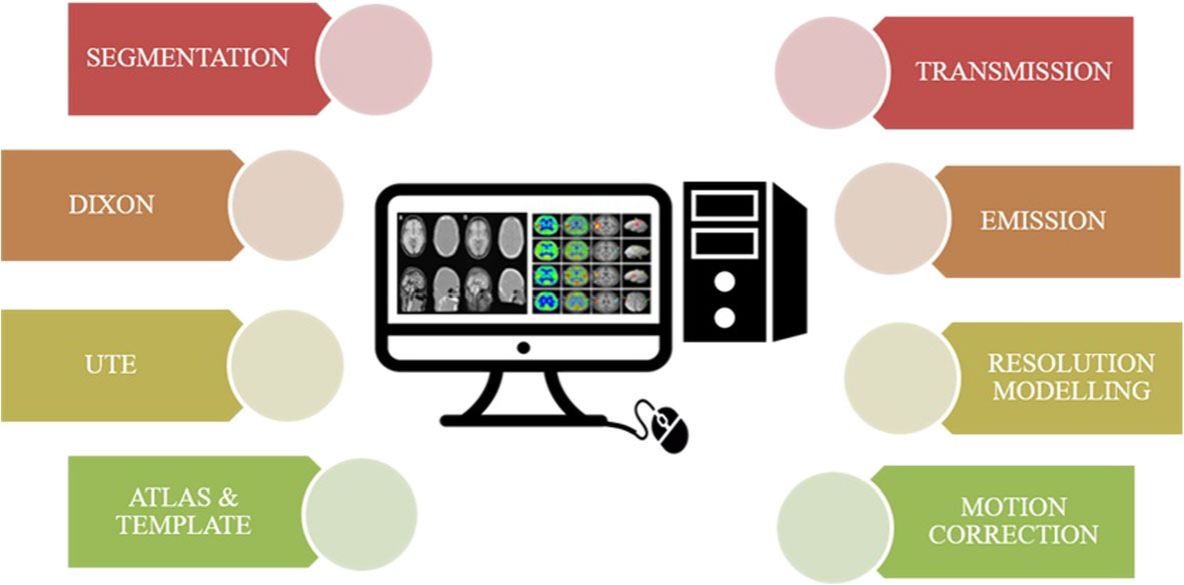
Various techniques of attenuation correction and reconstruction algorithms

Although various improvements have been made in the imaging modalities, data acquisition also involves pre-processing techniques such as image correction for obtaining the quantitative information from the image (Fig. 6) (Rahmim et al. 2007). The pre-processing techniques are supposed to be paradigmed to acquire the good quality of the image (Refer Table 3).

**Table 3:** Attenuation correction techniques

Technique	Merits	Demerits	References
Dixon (MRI)	● Distinguishes fat from water ● Localises uptake volume of PET ● Extracts the lung with segmentation	● Limited usage for attenuation correction ● Needs different echo times ● Cannot be used for detection of bone	(Martinez-Möller et al. 2009; Hu et al. 2009; Schulz et al. 2011)
UTE (MRI)	● Aids in detection of bone region	● Consumes extra time for MRI data acquisition ● Not applicable for whole body imaging	(Schulz et al. 2011; Keereman et al. 2010; Catana et al. 2010)
Atlas &Template (MRI)	● Faster process ● Minimal or no dosage is required	● Causes anatomical abnormalities ● Not applicable for whole body imaging ● Certain FOV gets truncated ● Requires templates for coils	(Hofmann et al. 2008; Kops and Herzog 2013; Malone et al. 2011; Klein et al. 2010)
Emission (PET)	● Minimal or zero additional acquisition time. ● Uses TOF information also.	● Less response to radionuclides such as FDG ● Requires templates for coils	(Nuyts et al. 1999; Defrise et al. 2012; Rezaei et al. 2012; Boellaard et al. 2014)
Transmission (PET)	● Primarily used for analysis of field of view	● Needs additional dose of radionuclides ● The attenuation map is corrupted with noise ● The spatial resolution is minimal	(Berker et al. 2014; Mollet et al. 2012; Mollet et al. 2012; Mollet et al. 2014; Watson et al. 2013)

### Attenuation correction in PET/MRI

The degradation of the PET images is mainly due to the scattering and attenuation of 511 K eV gamma rays within the FOV (Abella et al. 2007). The degree of attenuation and accuracy of attenuation mapping is based on the volume of the object (Zaidi et al. 2006). However, in case of PET-MRI, the attenuation due to hardware and MRI coils within FOV is near to the region of interest. Thereby, this can suppress the information from the PET image (Keereman et al. 2013). Any attenuation correction technique has to consume minimum time because the mapping is done simultaneously with acquisition of PET data (Zaidi and Hasegawa 2003).

#### MRI based attenuation correction

The advantage of co-registration of PET data on the structural image of MRI allows the attenuation correction simultaneously with the acquisition of PET image (Wollenweber 2002; An et al. 2015). The various techniques are discussed as follows.

**Fig. 7:**
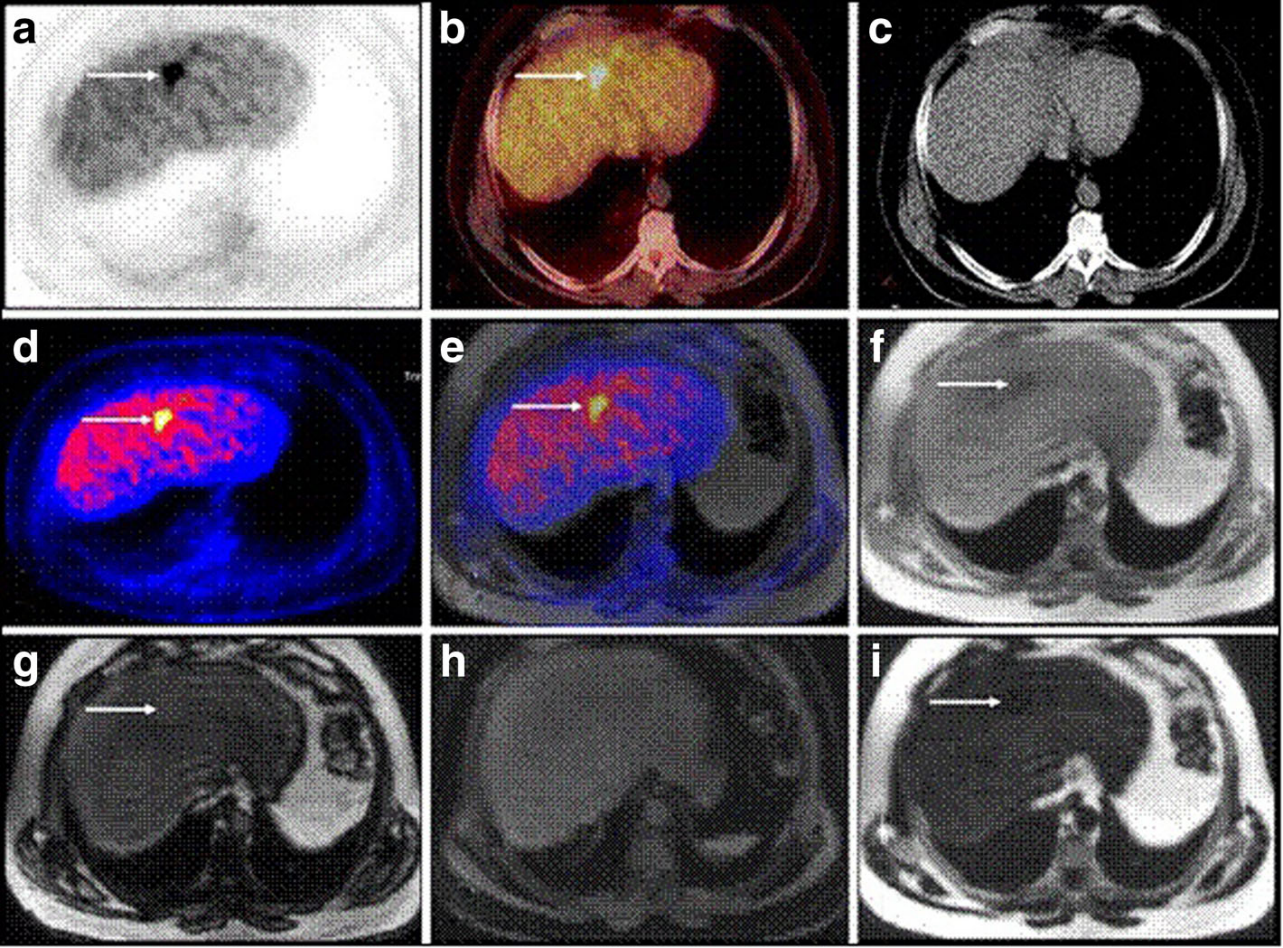
Set of images in a 48-year-old patient with a liver metastasis from sigmoid cancer. High liver uptake is found both in PETAC_CT (**a**) and PETAC_MR (**d**), whereas image fusion with low-dose CT (**b**) and Dixon T1w (**e**) allow for better anatomical correlation. In low-dose CT due to its low soft tissue contrast no anatomical correlate for the liver metastasis could be found (**c**). Figures from (**f**) to (I) present the different sets created from the raw data of the Dixon sequence ((**f**) T1w in-phase, (**g**) T1w out-of-phase, (**h**) water-only and (**i**) fat-only). The complementary value of different reconstructions can be appreciated as the liver metastases are outlined with different quality in T1w in-phase (**f**), T1w out-of-phase (**g**) and fat-only (**i**).No correlate can be found in the water-only image (**h**) (This research was originally published in *M. Eiber* et al., “Value of a Dixon-based MR/PET attenuation correction sequence for the localization and evaluation of PET-positive lesions, (Eiber et al. 2011)” Eur. J. Nucl. Med. Mol. Imaging, vol. 38, no. 9, pp. 1691–1701, 2011.© by the Society of Nuclear Medicine and Molecular Imaging, Inc.)

*Dixon* based MR sequence has the potential to distinguish between adipose and water-based tissues. The difference in the larmour frequency of protons present in water and fat can be utilised as distinguishing factor for MRI images acquired with different echo pulses (Martinez-Möller et al. 2009). In an experiment for evaluating the Dixon sequence used in anatomical correlation of PET-positive lesions, the sequence could replace the function of a low-dose CT in an MR/PET scanner in many applications besides its application in attenuation correction (Fig. 7). Thereby, it minimizes the requirement for the other sequences in areas of the body that cannot be covered by fully diagnostic MR sequences (Eiber et al. 2011).It helps to provide anatomical information co-registered with localisation of PET uptake (Hu et al. 2009). However, this method cannot be used for detection of bones though it allows segmentation of lungs from the image of low intensity (Schulz et al. 2011). But in recent study, a new hybrid ZTE (Zero Echo time) /Dixon MR-based attenuation correction (MRAC) method including bone density estimation for PET/MRI is performed (Leynes et al. 2017). It helps to quantify the effects of bone attenuation on metastatic lesions uptake in the pelvis. The water- and fat-suppressed projection imaging (WASPI) method utilises a ZTE sequence that includes fat and soft tissue suppression for generating the continuous-valued bone attenuation coefficients. As the bone density is found to be inversely proportional to the ZTE signal intensity, this method enables the quantification of bone, which cannot be performed with a typical Dixon sequence. The hybrid ZTE/Dixon pseudo CT is obtained by combining the Dixon continuous-valued fat and water pseudo CT and the ZTE-derived continuous-valued bone HU. It is depicted with the function.

HybridHU(x) = ZTEbone(x), Bonemask(x) = 1.

DixonHU(x); otherwise.

ZTEboneHU(x) = ZTE derived continuous valued HU.

Bonemask(x) = segmented bone region.

DixonHU(x) = Dixon derived HU values.

**Fig. 8:**
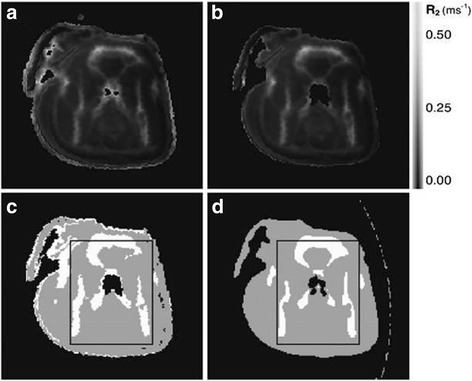
Transverse slices of uncorrected (**a**) and corrected (**b**) R2 maps and segmented MR (**c**) and CT (**d**) images of phantom (This research was originally published in*V. Keereman, Y. Fierens, T. Broux, Y. De Deene, M. Lonneux, and S. Vandenberghe*, “MRI-based attenuation correction for PET/MRI using ultrashort echo time sequences, (Catana et al. 2010)” *J. Nucl. Med.*, vol. 51, no. 5, pp. 812–818, 2010.© by the Society of Nuclear Medicine and Molecular Imaging, Inc.)

Thus, the shortcomings of Dixon can be overcome by using Ultra-Short Echo time (UTE) sequence. These sequences are used to observe the tissues with faster T_2_ relaxation time. It provides the image of the bone with sufficiently high intensity. This attenuation correction technique is based on the acquisition of MRI signal for two different echo pulses. This MRI sequence is also called as Dual-echo Ultra-Short Echo time (DUTE), when a couple of echoes are used for the acquisition of single image. In this, the image obtained at first echo (TE1 = 70-150 μs) is used to estimate the bone tissue with the discrete fast induction decay(FID) signal, whereas, the image at second echo (TE2 = 1.8 ms) contains no bone image as it is a gradient echo image (Keereman et al. 2010). R2 mapping is performed with the reciprocal of spin-spin relaxation time (T2) from the couple of images obtained in it with DUTE sequence as demonstrated by *Keereman* et al. This R2 map enables to distinguish soft tissue from cortical bone. Also, region growing technique is used for framing a binary mask from the TE1 image that could be used to mask the noise for clear cut difference between the soft tissue and air (Fig. 8). Eventually, the segmented regions are marked with the attenuation coefficientsc. Following it, *Catana* et al. performed morphological open and close operations to segment the head from the external voxels. The close filter masks the soft tissue, whereas the open filter is appliedto the image obtained at second echo (TE2) (Catana et al. 2010). Hence, further smoothening and normalising of the difference image is used to enhance the tissues of bone. Also, the detection of bone can be done along with distinguishing between fat and water by using UTE triple echo (UTILE) MR sequence combined with Dixon sequence as proposed by Berker et al. (2012) in his demonstration of four class (bone, soft tissue, fat tissue and air) segmentation. Adding to it, *Johansson* et al. also developed a complete voxel based method to produce a pseudo-CT, known as substitute CT (s-CT) with Gaussian regression model (Johansson et al. 2011). The generated s-CT provides the continuous attenuation coefficients as performed in the method of *Hofmann* et al (Hofmann et al. 2008).These sequences are utilised only for brain imaging because it consumes quite a long time, typically 3–5 min for every position of the bed. Due to its prolonged acquisition time, it introduces artefacts in the image acquisition.

*Atlas* is the method which depends on the existing MRI-CT data sets of the atlas. It employs the knowledge-based and machine-learning techniques (Hofmann et al. 2008). The MRI images of the atlas will be registered on the measured MRI image of the subject. This approach provides the structural space using μ-map or pseudo CT template which will be transformed to superimpose over the MRI of the subject (Kops and Herzog 2013). An experiment was performed on the patients of dementia disorder to compare the four different novel methods of atlas-based attenuation correction method for framing μ map from MRI image on the basis of tissue classification, quantitative accuracy of reconstruction and precision of the image. In the four different methods, two of them are atlas based method, one of them is segmentation based method and the other is of hybrid atlas/segmentation method. It was observed that the novel methods produced less error compared to that of the vendor’s AC method (Cabello et al. 2016). *TetsuroSekine* et al. has also compared that sequence based attenuation correction using ZTE with Atlas based attenuation correction for Brain 18F-FDG PET/MRI. Although, it was observed that ZTE-AC has more accurate results that Atlas-AC with enhanced head-skull attenuation, it causes mismatching in mastoid and nasal regions (Sekine et al. 2017). He also stated that Atlas-AC has more accuracy than that of CT based AC (Pet et al. n.d.). In a recent study, *Jenkins* et al. evaluated the accuracy of ATAC for brain PET in a TOF-PET/MRI system. The reconstruction of PET image is obtained with four different attenuation correction such as CTAC, PET with ATAC (air and bone are from an atlas), PET with ATAC_patientbone_(air and tissue are from the atlas accounting for patient bone) and PET with ATAC_boneless_(air and tissue are from the atlas not accounting for patient bone). However, it was observed that ATAC in PET/MRI acquires the same amount of quantification accuracy as that of CTAC in terms of bone compensation. Due to the attenuation differences based on anatomic variations it results in mismatching sinuses (Jenkins et al. 2017).

**Fig. 9:**
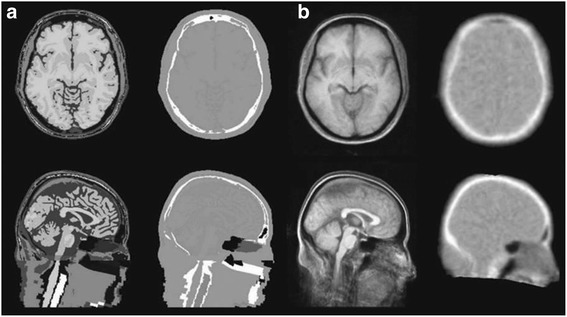
Transverse and sagittal sections through MRI and attenuation maps generated from the tissue atlas (**a**) and measured templates (**b**) (This research was originally published in *I. B. Malone, R. E. Ansorge, G. B. Williams, P. J. Nestor, T. A. Carpenter, and T. D. Fryer*, “Attenuation correction methods suitable for brain imaging with a PET/MRI scanner: a comparison of tissue atlas and template attenuation map approaches., (Malone et al. 2011)” J. Nucl. Med., vol. 52, no. 7, pp. 1142–9, 2011. © by the Society of Nuclear Medicine and Molecular Imaging, Inc.)

Similarly, *Template* sequence can be achieved in multiple methods, but the most reliable way is to combine the CT scans or PET transmission scans. This can be interpreted as averaging of the database of scans with detailed structure of MRI image. This technique has a potential to deliver wide structural information which can be segmented and labelled for analysis (Fig. 9) (Malone et al. 2011). However, with respect to PET based attenuation correction, template based attenuation correction (TBA) is a promising alternative as it reconstructs the radioactivity up to 9% (Kops and Herzog 2007). Also, *Rota Kops* et al. suggested that gender related TBA-f&m offers significantly similar results for both the genders (Kops and Herzog 2008). It can be used for brain imaging as it accounts the whole anatomical variations in the body but it cannot be used in the regions of minimised anatomical differences (Klein et al. 2010).

#### Pet based attenuation correction

An alternative approach to the MRI based attenuation correction is to extract the attenuation information from the PET data due to its shorter scanning time over MRI. There exist several techniques for it. However, it has been broadly classified into two categories as discussed below.

*Transmission* imaging is widely used for standalone systems of PET imaging as it provides the linear attenuation coefficient at 511 k eV or closer to it. It is also used to determine the linear attenuation coefficient of the object within the FOV which cannot be obtained in MRI based methods (Mollet et al. 2012). However, there exist certain challenges with this method such as noisy transmission images due to limited statistics, count rate of PET modality, smallness of the bore size of the PET/MRI modality compared to that of standalone PET system, longer acquisition time and crossover between the transmission and emission data. Thereby, a faster sequential transmission scan is required. Also, the utilisation of TOF information can rectify the cross over between transmission and emission data. This technique has been proved using simulations and experimental setup on annulus transmission source (Mollet et al. 2012). It delivers that adequate information can be derived for attenuation mapping using iterative reconstruction methods. It is applicable only for systems with TOF information such as Philips TOF PET/MRI and GE TOF-PET/MRI (Mollet et al. 2014). The utilisation of scatter details for attenuation mapping is proposed by the group in Aachen (Berker et al. 2014). The another approach of utilising magnetic field in MRI to inject the beams of positron from external emitting source without any shielding is proposed by Watson et al. (2013). This is an interesting technique as it uses a compact transmission source. As the transmission source is formed from the positron beams being obstructed by the material in the FOV.

On the other hand, *Emission* method utilises certain algorithms such as Maximum Likelihood reconstruction of Attenuation and Activity (MLAA), which has been introduced before the proposal of integrated PET/MRI modality. With the uniformity of the non-attenuated data one can extract the attenuation sinogram of the emission values (Nuyts et al. 1999). However, it has been observed that emission data alone is not enough for determining the attenuation correction factor. Hence, the TOF information can be a unique solution for attenuation (Defrise et al. 2012) and emission (Rezaei et al. 2012). Thereby, it is followed in PET/MRI modality as well. In the regions of truncation, a large attenuation mapping is done by utilising the emission values for fixing the missing data. MLAA algorithm is utilised to obtain a good estimate of truncated arms and shoulders. MLAA algorithm can also be used to obtain the attenuation map from the emission data of PET. Thus, this enables the whole body imaging in the presence of metal implants (Fuin et al. 2017). As it has the integrated non-TOF PET/MRI scanner, it could perform the implant PET based attenuation map completion (IPAC) method for combining the reconstruction of radioactivity and emission data to estimate the location, shape and linear attenuation coefficient of the metallic implant. However, this technique has many limitations associated with it, such as inability to have uptake of radiotracers in all the regions for determining the attenuation coefficients of the various regions, attenuation of materials outside the body that do not possess emission and complexity of scatter correction introduces cross over between emission and attenuation data (Boellaard et al. 2014).

### Reconstruction techniques in PET/MRI

The improvement of PET images in PET/MRI can also be achieved to produce high resolution anatomical data. It also aids in rectification of motion artefacts in MRI images to enhance the quality of the PET image (der Kouwe et al. 2006). The two commonly utilised techniques are:

#### Resolution modelling

The partial-volume effect (PVE) occurs as a consequence of limited spatial resolution. This phenomenon can be rectified using several methods in PET image (Rousset et al. 2007; Meechai et al. 2015). Thereby, reconstruction through PET can be modified and improvised using the details obtained from the structural image of MRI (Müller-Gärtner et al. 1992). As a fact, the high resolution in brain imaging can be achieved through co-registered PET and MRI in simultaneous acquisition system (Vunckx et al. 2012). The different methods to enhance the reconstruction of PET image from the information delivered by MRI is discussed by Bai et al. (2013). However, the technique has to be considerate over the non-isotropic distribution of positron annihilation due to the presence of magnetic field. This can be seen in the edges of lungs in the reconstructed images.

#### Motion correction

The introduction of motion artefacts corrupt the resolution of the PET image (Ouyang et al. 2013). It could be introduced by either rigid motion or non-rigid motion. The rigid motions are due to translation and rotation, namely head movement and body adjustments during acquisition. On the other hand, non-rigid movements are due to changes in shape, namely breathing artefacts (Chun et al. 2012). Although the movement of the patient can be constricted, non-rigid motions can be suppressed using gating or other motion correction methodologies.

**Fig. 10:**
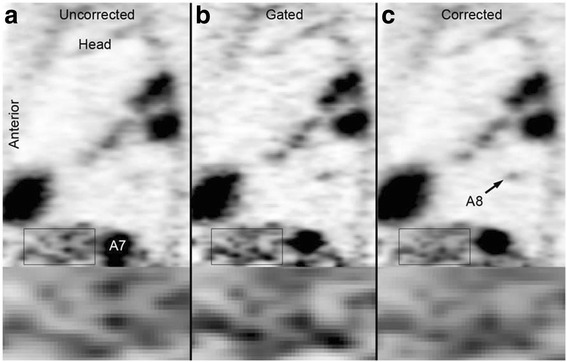
Comparison of uncorrected (**a**), gated (**b**), and corrected (**c**) sagittal PET image slice featuring lesions A7 and A8 in patient A. Multiple lesions and inflammatory areas with increased 18F-FDG uptake in lung show enhanced delineation (This research was originally published in *Würslin C, Schmidt H, Martirosian P, Brendle C, Boss A, Schwenzer NF,* et al., “Respiratory motion correction in oncologic PET using T1-weighted MR imaging on a simultaneous whole-body PET/MR system, (Würslin et al. 2013)” J. Nucl. Med. Soc Nuclear Med; 2013;54:464–71. © by the Society of Nuclear Medicine and Molecular Imaging, Inc.)

In gating, the data from the list-mode are separated as respiratory or cardiac phase. The signal to noise ratio depreciates in the resulted images. To prevent this, techniques that keep the count for all states by transforming them into a reference gating state have been used. The transformation can be done only after the reconstruction process is finishes. Hence, the images from different gating states are deformed to a reference gating state and cumulated (Dikaios et al. 2012) either during (Chun et al. 2012) or before the reconstruction techniques (Catana et al. 2010; Würslin et al. 2013). The transformation is performed using the deformation fields, which is derived from the PET data through co-registration of reconstructed gating states. Unfortunately, the approach is limited due to lack of anatomical information from PET data. However, the deformation fields are extracted from MRI data in simultaneous PET/MRI acquisition systems. To achieve respiratory gating, signals can be derived from the navigator images (Fig. 10) (Würslin et al. 2013), while for cardiac gating is done using a trigger single based on electrocardiography (ECG) (Büscher et al. 2010). Also, self-gated cardiac imaging is developed for simultaneous acquisition PET/MRI modalities (Larson et al. 2004). The various motion correction techniques using the information from the MRI data are tagged MR sequences (Boellaard et al. 2014) dimensional gradient echo images (Jenkins et al. 2017). Also, it is used for deriving the motion estimates from embedded clover leaf navigator (Pet et al. n.d.).

### Segmentation in PET/MRI

An approach of discretizing the structural image (Berker et al. 2012). It enables the identification of different regions and classification of tissues based on the sequence of pulse used in MRI. Besides the availability of the shortcomings of this method, it also incorporates the improper labelling of regions and inability to account for the attenuation values as it keeps changing based on the thickness of tissue (Keereman et al. 2013). Also, the MRI data cannot be used directly for attenuation correction as it cannot provide the linear attenuation coefficients (mu maps), unlike CT. Therefore, these effects can be quantified by segmenting the attenuation map containing Standardized Uptake Values (SUVs). In an experiment of tissue classification for whole body PET/MRI in to four classes such as background, lungs, fat and soft tissue with comparison to that of PET/CT data, the attenuation map for CT data is obtained by bilinear transformation. The CT based attenuation map is based on intensity of voxels. It was observed that the utilisation of MRI-based attenuation map resulted in average SUV change of 2.3% for 6 lymph node metastases compared to the results obtained with CT- based attenuation correction (Martinez-Möller et al. 2009). The different techniques to perform segmentation can be from simple thresholding to complex morphological operations. The greatest advantage of this technique is that it can account for different shapes and positions of organs in the body, while the disadvantage is that the identification and classification of tissues are not easy in real scenario. Sometimes, due to partial volume effect, there could be a mismatching in identification of tissues. A segmentation approach using fuzzy clustering to segment the T1 weighted images into nasal sinus, skull, brain tissue and air with morphological operations was performed by Zaidi et al. (2003). Also, *Rota Kops* et al. utilised BrainSuite (Shattuck and Leahy 2002) and MPITool (Advanced Tomo Vision GmbH, Kerpen, Germany) for classifying the tissues such as cavity, bone, brain and soft tissue on the basis of attenuation coefficient (Kops and Herzog 2007). Also, *Wagenknecht* et al. used the knowledge-based post-processing approach for segmenting the extra-cerebral region from brain. This approach employs neutral networks for differentiating the grey and white matter, adipose tissue and cerebrospinal fluid and background (Wagenknecht et al. 2009; Wagenknecht et al. 2010; Wagenknecht et al. 2011; Kops et al. 2009).

### Data analysis and visualisation

Even though PET/MRI can produce images of high quality and resolution, it requires registration of images after acquisition along with the post-processing techniques. Thus, the degree of registration is based upon the registration of interest. For instance, it can be easier to do for brain image over whole-body image, as the former requires only linear transformations while the latter involves complexity. However, a good image should be obtained with no chance of repositioning the subjects for scanning (Veit-Haibach et al. 2013; Schmid et al. 2013).

**Fig. 11:**
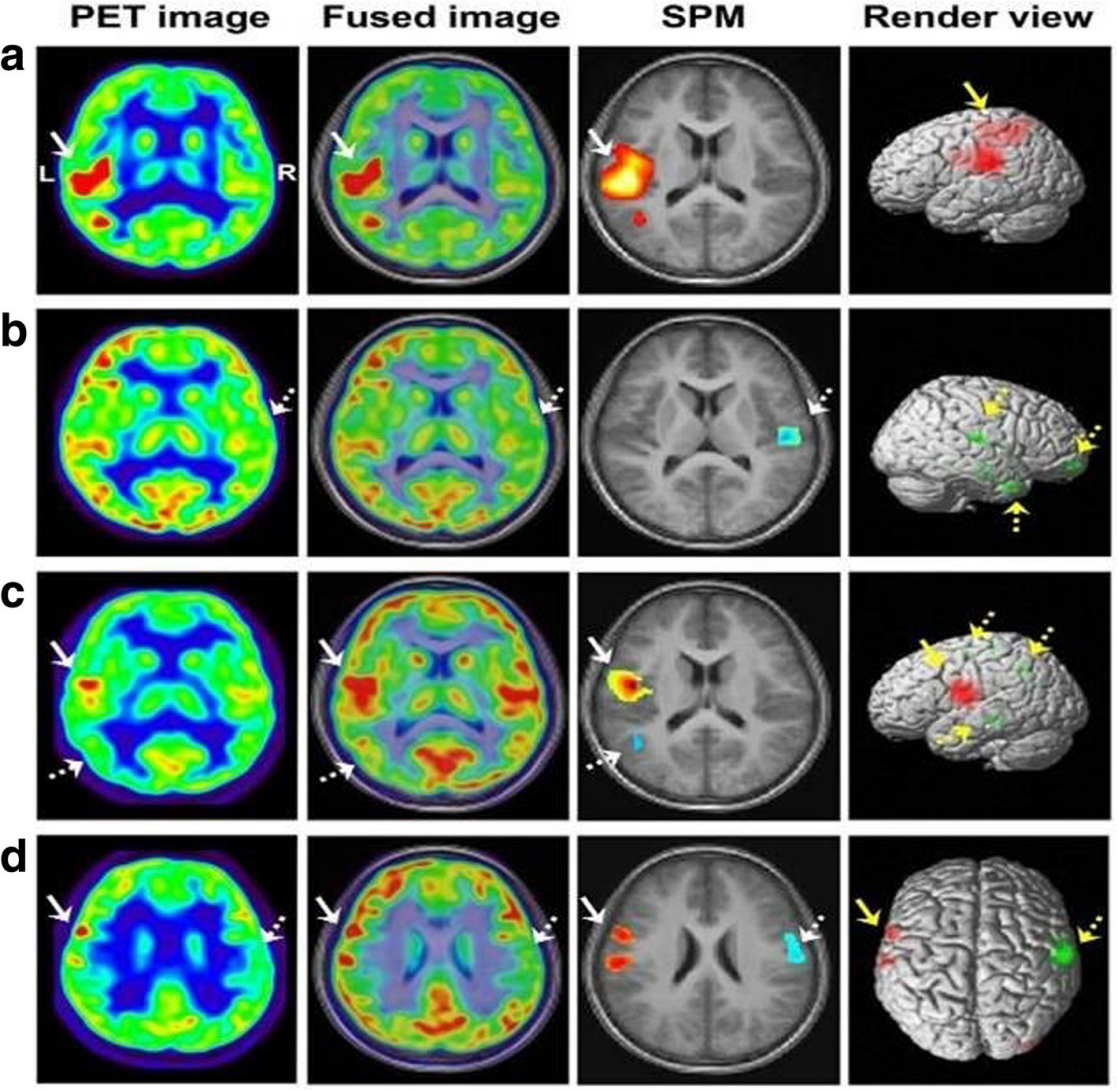
Comparison between visual and SPM analysis of 18F-FDG PET images. (**a**) Hypermetabolism in bilateral frontal lobes (solid arrow) was detected by both visual and SPM analysis. (**b**) Hypometabolism in left temporal lobe (solid arrow) was detected by both visual and SPM analysis. (**c**) Hypometabolism was found in left rolandic area (dashed arrow) by visual assessment, and hypermetabolic region was further identified in right rolandic area (solid arrow) by SPM analysis. (**d**) Hypometabolic region undetected by visual assessment was identified in left mesial temporal lobe (solid arrow) by SPM analysis. (This research was originally published in Y. Zhu et al., “Glucose Metabolic Profile by Visual Assessment Combined with Statistical Parametric Mapping Analysis in Pediatric Patients with Epilepsy, (Zhu et al. 2017)” J. Nucl. Med., vol. 58, no. 8, pp. 1293–1299, 2017.© by the Society of Nuclear Medicine and Molecular Imaging)

Earlier, it was believed that the information from MRI image can be used for defining the region of interest in PET data (Evans et al. 1988). However, Atlases are used to map certain regions that can be registered to the subject’s organ. Also, the atlases of human body are employed for determining the bio-distribution of tumours and metastases. The multimodal atlas can be acquired from a 3D slicer module for simple evaluation of the available image. This module has been validated with a publicly available soft tissue sarcoma information from the Cancer Imaging Archive (Rackerseder et al. 2017). Beyond this visualisation technique, retrospective analysis of structural and metabolic neuro-imaging can be performed using the free access software such as MRIcron, BrainSuite, BioImageSuite, ImageJ, FSL, Amide and MeVIS Lab. Usually, BrainSuite is used for stripping off the skull and creating the binary cerebral volume mask, hence, it will be processed with FSL FAST software. Also, the co-registration techniques can be achieved using BrainImageSuite. In recent time, *Yuankai Zhu* et al. evaluated the glucose metabolism in epileptic paediatric patients with the visual assessment and Statistical Parametric Mapping (SPM) (Fig. 11) (Zhu et al. 2017). While SPM is designed to perform segmentation of brain tissues consisting of Grey Matter (GM), White Matter (WM) and Cerebrospinal Fluid, the FSL and Brainsuite can segment sub-cortical structures also. Also, the performance of FSL was influenced by image noise and intensity non-homogenity (Kazemi and Noorizadeh 2014). Also, PMOD (version 3.5, PMOD Technologies, Zurich, Switzerland) was used in parametric images of Dopamine receptors (D_1_R) distribution volume ratio (DVR) and binding potential (BP_ND_) of the PET data from the multi-linear reference tissue model (Kaller et al. 2017). Even SPM8 was used for motion correction in this experiment. Also, this software was used in a research work of facilitating the PET imaging of ischemic heart disease with polyglucose nanoparticles to delineate the ROIs belonging to the T1 weighted image of the cardiac blood pool. The version used in this work was PMOD 3.4 (Keliher et al. 2017). The PMOD Analysis software is also used in calculating the SUV in the tumour bearing mice. The analyses were done using the viewing and fusing tools of the PMOD Analysis Software (Busk et al. 2017). Thus, this software can be used to evaluate the retention of tumour FDG in the control mice on various days. It also provides various other analyses such as histogram of voxel frequency distribution as it provides reproducibility over SUV based quantification. Scanner and reconstruction parameters can significantly affect SUV measurements. When using serial SUV measurements to assess early response to therapy, imaging should be performed on the same scanner using the same image acquisition and reconstruction protocols. In addition, attention to detail is required for accurate determination of the administered radiopharmaceutical dose (Adams et al. 2010). To overcome these shortcomings, machine learning approaches and medical informatics are employed to improvise the data analysis of PET/MRI modality. These techniques could be used to determine the irregularities in multimodal and multi-parametric imaging. Nevertheless, there exists a twin challenge, namely, making the neural models to be subject specific and calibrating the model parameters (Lan et al. 2016). Thus, the upcoming technology is anticipated to model the robust and reliable systems for computation.

### Challenges & future directions of PET/MRI

Beyond the promising aspects of the hybrid PET/MRI modality, there exists many challenges due to the technical glitches which have to be resolved before the utilisation for clinical applications (Brix et al. 2009). Thereby, effective research is essential for simultaneous PET/MRI with software based co-registration technique. This could compensate over the complex operation and will be cost effective too. The need for cost effective simultaneous and sequential system are needed (Martinez-Möller et al. 2012). Although PET/MR is costlier than PET/CT, it can perform simultaneous data acquisition and high probability of detecting low grade lymphomas. In case of sequential design mode of PET/MR, it has the tendency to incorporate motion artefacts of organs and the patients or subjects are instructed to switch the couch every time for different modality scanning. Adding to this, it also occupies humongous area for two different separate modalities. However, there had been numerous technical glitches when they share the same gantry as discussed in the main section. Also, genotoxic potential of ionizing radiation increases in low frequency static magnetic field (Koyama et al. 2005; Miyakoshi et al. 2000; Walleczek et al. 1999; Hintenlang 1993). Therefore, a lot more research must be conducted to evaluate the radio nuclides of PET with MRI. The delineation of anatomic structure occurs due to very high level of magnetic field strength around 7 T. Thus, to enhance the fMRI technique efficacious research is going on for cost effective system (Judenhofer et al. 2007; Theysohn et al. 2008). The dosage of radiations has to be assessed in comparison to that of PET/CT. Besides Multimodal imaging, to better understand the disease and develop treatment strategies it requires a large number of biomarkers from various modalities including molecular, morphological, genotyping, fluid biomarkers and clinical assessments (Jin et al. 2016). Thus, the efficacious field of nanotheranostics have provided the wide opportunities for tagging the multimodal biomarkers, especially for targeting clinical pathologies. The notion of complementary information is acquired through the cocktail of imaging agents to assess simultaneously the two parameters about the pathology. For instance, ^18^F-FDG is tagged with Gd-DTPA to obtain the molecular information of the metabolism of a tissue, whereas the use of Gd-DTPA (MR contrast agent) provides the perfusion of the tissue (Vecchione et al. 2017).Although it could synergistically combine the imaging properties of PET/MR, it possesses the functional and synthesis limitations. These bimodal agents will be required in nanomolar concentration of PET contrast agent and millimolar concentration of MR contrast agent for higher sensitivity. This enormous difference makes the tagging of MR reporters to radiotracers unrealistic. Nevertheless, the detection of both the imaging signals with very low concentration of radiotracers is feasible when the radiotracers are tagged to the MR reporters (Poulin et al. 2013). Probing these various interfaces allows the development of predictive relationships between structure and activity that are determined by nanomaterial properties such as size, shape, surface chemistry, roughness and surface coatings (Nel et al. 2009). For instance, Cationic particles or particles with high surface reactivity are more likely to be toxic than the larger relatively hydrophobic or poorly dispersed particles, which are rapidly and safely removed by the reticuloendothelial system (RES) (Brannon-Peppas and Blanchette 2012). Also, particles that promote enhanced permeation and retention (EPR) effects and are therefore optimal for chemotherapeutic drug delivery to cancers, generally have mid-range sizes and relatively neutral surface charges (Acharya and Sahoo 2011). Thus, the biomolecular/ pharmaceutical kinetics of the nanoprobes have been a major issue in the clinical research such as penetration of Blood Brain Barrier (BBB). To address this limitation, the “Virtual Experimentation” module is developed, which allows the users to examine the effect of biochemical/pharmacokinetic parameters on tissue tracer kinetics through computer simulations (Huang et al. 2005). Besides, these multimodal feature extractions must be specialised with artificial intelligence and neural network modules. In addition to low sensitivity and signal-to-noise (SNR) of MRI, the technical and temporal limitations make it difficult to perform whole-body MRI. Thereby, radionuclide signal of bimodal agents could be used to detect areas of low uptake or outside the MRI field of view to perform high-resolution MRI (Rosales 2014). Nextly, the higher time consumption of whole body imaging over organ-specific imaging has been critical challenge, yet. However, the fast multiple organ detection and localization in Whole-Body MR Dixon Sequences enables the improvised anatomy localization accuracy with higher efficiency and robustness (Pauly et al. 2011).

Also, the protocol for injection of radiotracers and image acquisition parameters should be evaluated for optimised imaging of patients. The safety guidelines for procedural clinical applications in quality control and safety system are needed. The physicians, medical physicist, technologists and operators are to be certified by the bodies such as American College of Radiology and Society of Nuclear Medicine. Eventually, the guidelines of Medicare reimbursement must be obtained before clinical trials. Thus, the various safety and ethical related issues pertaining to PET/MR are supposed to be fixed for acquiring the enhanced applications in various fields of research.

## Conclusion

PET/MRI delivers the potent for ‘one stop shop’ combination of anatomical, metabolic and molecular imaging, which turns out to be better than PET/CT or stand-alone PET system. The post data acquisition including attenuation correction and reconstruction techniques are not sufficient to derive to the perfect results from it. Also, the shortcomings of the present system must be approached with brilliant notions to frame the better system. Further, new software based technologies must be developed for the clear-cut inference of the diseases. The system produces a better performance in terms of detection, practicality and technical feasibility. It is also a cost-effective system, for being affordable by the buyers. It adds up to the absolute merit of reducing the need of separate scanning for PET and MRI alone. Adding to the fact, PET stability metrics demonstrated that PET quantitation was not affected during simultaneous aggressive MRI. This stability enables demanding applications such as kinetic modelling (Deller et al. 2018). The evolvement of PET/MR has gross applications in the field Neuroscience, Oncology, Musculoskeletal, etc., thus, turning out to be a frontier in the era of complementary hybrid imaging. Yet, it also requires further investigations on the various other applications of PET/MRI for pre-clinical and clinical trials.

## Abbreviations

APD: Avalanche Photodiode
CT: Computed Tomography
DWI: Diffusion Weighted Imaging
fMRI: Functional MRI
G-APD: Geiger mode APD
McPET: MR compatible PET scanner
MMI: Multi-Modality Imaging
MPPC: Multi Pixel Photon Counter
MRI: Magnetic Resonance Imaging
MRS: Magnetic Resonance Spectroscopy
PET: Positron Emission Tomography
PMT: Photo Multiplier Tubes (PMT)
PSPMT: position sensitive PMTs
PVE: Partial-Volume Effect
PWI: Perfusion Weighted Imaging
SiPM: Silicon Photomultiplier
UHF: Ultra High Frequency
UTE: Ultra-Short Echo time
ZTE: Zero Echo time
